# Supplementary material for: The genome sequence of the Treble Lines, Charanyca trigrammica (Hufnagel, 1766) (Lepidoptera: Noctuidae)
Source: Wellcome Open Res. 2025 Sep 18;10:520. [Version 1] doi: 10.12688/wellcomeopenres.24867.1 (PMC12501593; doi:10.12688/wellcomeopenres.24867.1)
Supplement: Peer review history

# Reviewer report: Reviewer response for version 1

**DOI:** 10.21956/wellcomeopenres.27391.r134013

**Published:** 2025-10-27

**Authors:** Arun Arumugaperumal

**Affiliations:** 1 Department of Biotechnology, Rajalakshmi Engineering College, Thandalam 602105, Chennai, Tamilnadu, India

The genome note describes the whole genome sequencing of *Charanyca trigrammica, *also known as the Treble Lines. The article has been written well, and it is very interesting to read. The assembly reported here is of size 546.43 Mb, and the mitogenome size is 15.44 kb. The assembly has been scaffolded into 31 chromosomal molecules. Since the authors have sequenced a male specimen, they could capture only the Z sex chromosome. If they had sequenced a female specimen, they could have captured the W chromosome also. Already published, Charanyca member has the ZW sex system [1]. BUSCO completeness of 99% indicates that the genome sequence reported is a near-complete one. The annotation from Ensembl has not been released yet.

 In Table 1, the run accession ERR14209139 was given. But the text does not describe any sequencing using Sequel Ile instrument or the inclusion of such data in the assembly process. I can only assume that data from both runs are pooled together, from the number of bases mentioned in Table 1. Since this article is very nicely written and very appealing to the reader, anybody will naturally have the same doubt. Can the author include a line or two to clear this doubt?

Are sufficient details of methods and materials provided to allow replication by others?

Yes

Is the rationale for creating the dataset(s) clearly described?

Yes

Are the datasets clearly presented in a useable and accessible format?

Yes

Are the protocols appropriate and is the work technically sound?

Yes

Reviewer Expertise:

Bioinformatics; Genomics

I confirm that I have read this submission and believe that I have an appropriate level of expertise to confirm that it is of an acceptable scientific standard, however I have significant reservations, as outlined above.

## References

[1] : The genome sequence of the Brown Rustic, Charanyca ferruginea (Esper, 1785). *Wellcome Open Research* .2023;8: 10.12688/wellcomeopenres.20137.1 10.12688/wellcomeopenres.20137.1 PMC1131617839129912

# Reviewer report: Reviewer response for version 1

**DOI:** 10.21956/wellcomeopenres.27391.r134014

**Published:** 2025-10-06

**Authors:** Jhon Alex Dziechciarz Vidal

**Affiliations:** 1 Universidade Federal de SãoCarlos (UFSCar), São Carlos, Brazil

The manuscript presents the genome assembly of *Charanyca trigrammica*, describing the process of sample collection, species identification, tissue selection for DNA extraction, sequencing, and assembly. The background information about the species is valuable, as it helps readers better understand the evolutionary and ecological context of this moth. The methods are clearly and consistently described, and the level of detail is appropriate.

 I particularly appreciate the detailed description of how the species was identified, as morphological identification can be challenging for some taxa. The genome assembly methods rely on widely used tools, and since the pipeline follows the standards established by the consortium, it represents a consistent and reliable approach for genome assembly.

 The results are well presented, providing a thorough assessment of assembly quality, including Hi-C contact maps, figures, and tables that effectively illustrate the assembly metrics. The decision to assemble only haplotype 1 to the chromosome level was appropriate, considering that the specimen used was a male and therefore lacks heterogametic sex chromosomes to resolve.

 In conclusion, I have no concerns regarding the data report. The methodology and assembly quality meet current standards for genome assembly projects, and the manuscript represents a solid contribution to the field.

Are sufficient details of methods and materials provided to allow replication by others?

Yes

Is the rationale for creating the dataset(s) clearly described?

Yes

Are the datasets clearly presented in a useable and accessible format?

Yes

Are the protocols appropriate and is the work technically sound?

Yes

Reviewer Expertise:

My background is in cytogenetics, genetics, genomics, and bioinformatics, using different animal models, both vertebrates and invertebrates. Yes, I feel capable of assessing several aspects of this paper.

I confirm that I have read this submission and believe that I have an appropriate level of expertise to confirm that it is of an acceptable scientific standard.

# Reviewer report: Reviewer response for version 1

**DOI:** 10.21956/wellcomeopenres.27391.r134015

**Published:** 2025-10-03

**Authors:** Stephan Koblmüller

**Affiliations:** 1 University of Graz, Graz, Austria

In this manuscript, Boyes et al. present the whole genome assembly of the Treble Lines, *Charanyca trigrammica*, a noctuid moth that is widely distributed across Europe. Some background info about the target species is provided, which I appreciate. Also, the rationale for sequencing this particular species (part of a large whole genome sequencing initiative) is given, something that’s often forgotten in this kind of publications.

 The data are clear; the methods are well described. I have no objections to any of the data presented or the results. As far as I can tell, the datasets are deposited in publicly accessible repositories.

Are sufficient details of methods and materials provided to allow replication by others?

Yes

Is the rationale for creating the dataset(s) clearly described?

Yes

Are the datasets clearly presented in a useable and accessible format?

Yes

Are the protocols appropriate and is the work technically sound?

Yes

Reviewer Expertise:

phylogenetics/-genomics, population genetics

I confirm that I have read this submission and believe that I have an appropriate level of expertise to confirm that it is of an acceptable scientific standard.
